# A school-setting pilot study of the e-learning version of the “Journey of the Brave”: a universal anxiety-prevention program based on cognitive behavioral therapy

**DOI:** 10.1186/s13034-025-00956-8

**Published:** 2025-08-25

**Authors:** Shoichi Ohashi, Yuko Urao, Kazumasa Fujiwara, Takako Koshiba, Shin-ichi Ishikawa, Eiji Shimizu

**Affiliations:** 1 Research Center for Child Mental Development, Chiba University, 1-8-1 Inohana, Chuo-ku, Chiba, Japan; 2 United Graduate School of Child Development, Osaka University, Osaka, Japan; 3 Graduate School of Education, Hyogo University of Teacher Education, Hyogo, Japan; 4 Faculty of Psychology, Doshisha University, Kyoto, Japan

**Keywords:** Children’s anxiety, Universal prevention program, Internet-based cognitive behavioral therapy (I-CBT), E-learning, School setting, Gamification, Pilot study

## Abstract

**Background:**

School-based cognitive behavioral therapy (CBT) programs delivered by teachers are effective in preventing anxiety among children. Internet-based CBT has emerged as an efficient method for delivering such interventions. Our previous research demonstrated the feasibility of the e-learning version of *Journey of the Brave* in reducing anxiety among Japanese elementary school students in a home-based learning environment. This study aimed to evaluate the feasibility and acceptability of the e-learning version in school settings and to identify any practical implementation issues.

**Methods:**

We conducted a single-arm study with 204 elementary school students aged 10–12 years. Participants completed the e-learning program during regular classroom hours. Of these, 180 students who completed more than 80% of the program and agreed to participate in the study were included in the analysis. We evaluated changes in anxiety symptoms using the Spence Children’s Anxiety Scale (SCAS), alongside learning logs and a post-program satisfaction questionnaire.

**Results:**

The estimated mean SCAS score, analyzed using a generalized linear mixed model, showed a significant decrease of 2.6 points from pre-intervention to follow-up (*p* = .04, 95% CI [-5.08, -0.09]). Subgroup analysis revealed a 6.4-point reduction in boys compared to a 0.5-point decrease in girls. In School A, the SCAS score slightly increased by 0.4 points, while in School B, it decreased by 3.1 points. Significant interaction effects were observed between time and gender (*p* = .03) and between time and school (*p* = .04). The mean comprehension test score (true/false) was 13.2 out of 16 (SD = 2.2).

**Conclusion:**

The *Journey of the Brave* e-learning version was feasible and well-accepted in school settings. Student self-reports suggested that they may have applied the CBT knowledge and skills in their daily lives. Future studies should explore the contextual factors influencing program effectiveness and determine optimal conditions for its implementation.

*Trial registration*: UMIN, UMIN000057115, Registered February 21, 2025.

## Background

Anxiety disorders are among the most common mental disorders, with a prevalence of 6.5% (CI 95% 4.7–9.1) in children and adolescents, as reported in a study that integrated data from 27 countries [1]. Additionally, the prevalence of anxiety disorders across all age groups has increased by 25.6% (UI 95% 23.2–28.0) following the COVID-19 pandemic, increasing the need for improved mental health resources worldwide [2]. In Japan, the percentage of people suspected of having generalized anxiety disorder is 7.6%, while more than half (51.2%) have never visited a medical institution for mental health reasons, and 76.5% have never even heard of the term generalized anxiety disorder [3]. In a large-scale epidemiological survey conducted in the United States and Europe, up to 33.7% of the population experiences some form of anxiety disorder during their lifetime; the 12-month prevalence rate of anxiety disorders among adolescents aged 13 to 17 was 24.9%, with a median age of onset of 11 years [4]. Because approximately three-quarters of anxiety disorders originate in childhood, and children with anxiety disorders are 3.5 times more likely to experience depression and anxiety in adulthood [5], early intervention, including education to understand and prevent these disorders during childhood, is critical.

Cognitive behavioral therapy (CBT) is the gold standard treatment for childhood anxiety disorders. With a recovery rate of 47–66%, it addresses cognitive biases that contribute to anxiety and avoidance behaviors, as reinforced by learning theory, promoting gradual exposure to anxiety to improve symptoms [6]. However, few children with anxiety disorders seek help and access CBT, and school-based approaches have been implemented as an effective way for children to access treatment [7]. According to a meta-analysis, psychological or psychoeducational prevention programs conducted in schools that aim to address depression, anxiety, or overall mental well-being can reduce anxiety to some extent [8]. Several of these programs aim to prevent the onset of anxiety disorders, and many are universal programs that target everyone [7].

The Institute of Medicine classifies prevention interventions into three levels: (1) universal, (2) selective, and (3) indicated interventions [9]. Universal interventions, which are provided to everyone without targeting specific groups, can avoid labeling and stigma [10]; therefore, they are suitable for implementation in schools where children spend their time in groups. In Japan, the school-based anxiety-prevention education program “Journey of the Brave,” which is based on CBT and targets children in the fifth grade (age 10) and above, has repeatedly been reported to be effective in reducing anxiety scores among elementary school children even when implemented by teachers who are not therapists or other professionals [11–13]. The “Journey of the Brave” program is implemented as part of the school curriculum and consists of 8–10 classes that teach psychological education regarding anxiety, relaxation skills, the development of “anxiety stairs” for gradual exposure, the cognitive model and cognitive restructuring, and assertion skills. Although this approach is logistically advantageous because it can be implemented by the teachers themselves, the heavy workload of Japanese teachers presents a challenge. The Ministry of Education, Culture, Sports, Science, and Technology (MEXT) announced a comprehensive policy on work-style reform for teachers in 2019 [14], which has resulted in gradual improvements regarding the workload of teachers; however, teachers still spend more than 10 h a day at school [15]. The current Japanese education curriculum allocates almost no class time to mental health; thus, addressing mental health issues would require busy schools to add another educational activity to their already heavy workload. Thus, anxiety-prevention education needs to be provided in a less burdensome way.

To this end, we developed an e-learning version of the “Journey of the Brave” that allows children to learn online using computer devices. Internet-based CBT (I-CBT) is cost-effective [16] and is as effective as face-to-face CBT in treating children’s anxiety [17, 18]. Recent reports indicate that I-CBT programs designed to prevent anxiety and depression through transdiagnostic interventions have improved anxiety symptoms, anxiety sensitivity, and emotional avoidance [19]. Our previous study, which reported that the e-learning version of the “Journey of the Brave” was feasible for use when children learned at home to prevent their anxiety, although it was a single-arm study, showed a reduction in anxiety and an improvement in emotional regulation skills from before learning to follow-up [20]. In this study, we evaluated the feasibility and acceptability of the “Journey of the Brave” e-learning version when it is provided through schools to further contribute to anxiety-prevention education.

In the “Journey of the Brave” e-learning version, teachers do not need to implement the class in a conventional manner. Rather, students can use class time to learn at their own pace. Thus, teachers minimize the amount of time and effort they need to spend on preparation and learning support. This study was conducted to evaluate whether the use of the “Journey of the Brave” e-learning version as part of school learning activities would enable students to complete their learning independently, and whether it would be possible to acquire knowledge related to reducing and preventing anxiety. Additionally, Werner-Seidler et al. have noted the need for further studies to clarify whether the digital delivery of preventive intervention programs for depression and anxiety is a feasible long-term solution in schools, as the variety of programs is still limited despite the widespread use of digital delivery [8]. Therefore, we also assessed the practical issues of implementing the “Journey of the Brave” e-learning version in schools as a preparatory step toward realizing larger-scale implementation studies in the future, including randomized controlled trials.

## Methods

### Aim and setting

This study evaluated the feasibility, acceptability, and practical issues of implementing the e-learning version of “Journey of the Brave” [20] in a school setting. It was conducted as a single-arm intervention study without a control group. This study was conducted from September 2023 to March 2024 and was based on a research plan (Registration number: M10374) approved by the Ethics Review Committee of Chiba University.

### Participants and recruitment

The participants were 204 children in the fifth and sixth grades (aged 10 to 12 years) from two elementary schools in Chiba, a prefecture neighboring Tokyo. School A was a small school with only one class for each grade, and all students in the fifth and sixth grades participated. School B was a large school located near a major train station, and only fifth-grade students participated. The e-learning program was designed for anxiety prevention, specifically targeting children attending regular classes. Students enrolled in special needs classrooms who had apparent difficulties and were receiving individualized education plans or support were excluded. Although there was a possibility that some students in regular classes who frequently missed school or who had mental health issues or developmental disorders were among the participants, they were not excluded because they were participating in the same classes as usual, and the program was designed as a universal intervention. In recruiting participants, we posted the recruitment guidelines on the official website and recruited applicants through school units, local government boards of education that had implemented the “Journey of the Brave” class in person in the past, and teachers who had participated in teacher training workshops for the “Journey of the Brave.” The e-learning program presents low-risk preventive educational activities implemented independently by schools as part of their regular curriculum. Our involvement was limited to conducting anonymous questionnaire surveys both before and after the program. Therefore, we ensured that parents had the right to opt out of their children’s participation by providing written notification through the school and obtaining consent through an opt-out procedure. We requested that teachers not force children who did not consent to participate in the study to answer the questionnaire or provide data.

### Intervention

The “Journey of the Brave” e-learning version is a program that allows children to learn at their own pace online by watching videos, covering the same content that was originally taught in face-to-face classes. The eight learning stages and the summary stage are designed to be completed in nine class hours, with homework assigned between each stage. The program is designed with a reward feature that encourages children to learn enthusiastically, offering points when they complete the check tests for each stage and submit their homework. New items and characters are displayed on screen as they progress in their learning [20]. In this study, each student was given an e-learning account and logged in from their own computer devices following their homeroom teacher’s instructions. They watched videos and worked on assignments during class time. The e-learning platform included videos explaining how to proceed with learning and how to operate the system. However, teachers who had received training on how to conduct the “Journey of the Brave” class provided support to students during the class. Thus, regardless of individual issues such as unfamiliarity with computers, language barriers, or concentration difficulties, all students could learn through e-learning. Additionally, it was possible to divide one stage into smaller sections and progress through them during short homeroom activities (approximately half the length of a class hour). As e-learning can be accessed anytime and anywhere, children could complete unfinished work outside of class or learn later if they were absent. Conversely, teachers could also skip learning that had not been completed within the specified time owing to slow progress or absence while operating the program. We entrusted the homeroom teachers of each class with operating the program, which included scheduling class times for student access and deciding whether to follow-up with students who did not complete the program during class time or those who were absent, or to skip that part altogether.

### Measurements

The primary measurement of this study was the Spence Children’s Anxiety Scale (SCAS). The SCAS is a 38-item psychological scale that evaluates the severity of anxiety disorders in children [21]. Its factor structure, consisting of six subscales (separation anxiety, social phobia, obsessive-compulsive disorder, panic-agoraphobia, generalized anxiety, and fears of physical injury), has been supported by the systematic reviews of numerous studies [22]. The reliability and validity of the Japanese version have been confirmed (Cronbach’s alpha = 0.94) [23]. In previous studies that implemented the “Journey of the Brave” in face-to-face classes at elementary schools, a reduction in the mean score as a result of the intervention was reported [9–11]. In this study, we collected data at three time points: before the start of intervention (pre), at the end of intervention (post), and at follow-up (which occurred two to three months after the end of learning). As this is the first study to implement the “Journey of the Brave” e-learning version in a school setting, we also collected learning logs including homework (number of sessions completed, test scores, etc.), and the results of satisfaction questionnaires conducted using a four-point scale as secondary evaluation measures to assess feasibility, acceptability, and practical issues.

### Analysis

As this study evaluated the feasibility and acceptability of the program and to assess practical issues in its implementation in schools, we analyzed the data of students who completed at least 80% of the program. We used a generalized linear mixed model (GLMM) to analyze changes in SCAS scores over time and multiple comparison tests to assess differences between the three time points. The GLMM can estimate the score changes of the entire group, even if there are participants with missing data in the three questionnaire responses, thereby allowing the acquired data to be incorporated into the analysis without waste. In this study, we also conducted a subgroup analysis to examine differences by gender and school, in addition to overall score changes. Gender was self-reported as boy, girl, or other, and no response was also accepted. We used the Kruskal–Wallis and Mann–Whitney U tests to compare baseline (pre) scores. Regarding the evaluation of feasibility and acceptability, we conducted a comprehensive assessment based not only on changes in scores, but also on the results of the learning log data and satisfaction questionnaires. Statistical analyses were performed using SPSS Statistics version 28 (IBM, Armonk, NY, USA).

## Results

**Fig. 1:**
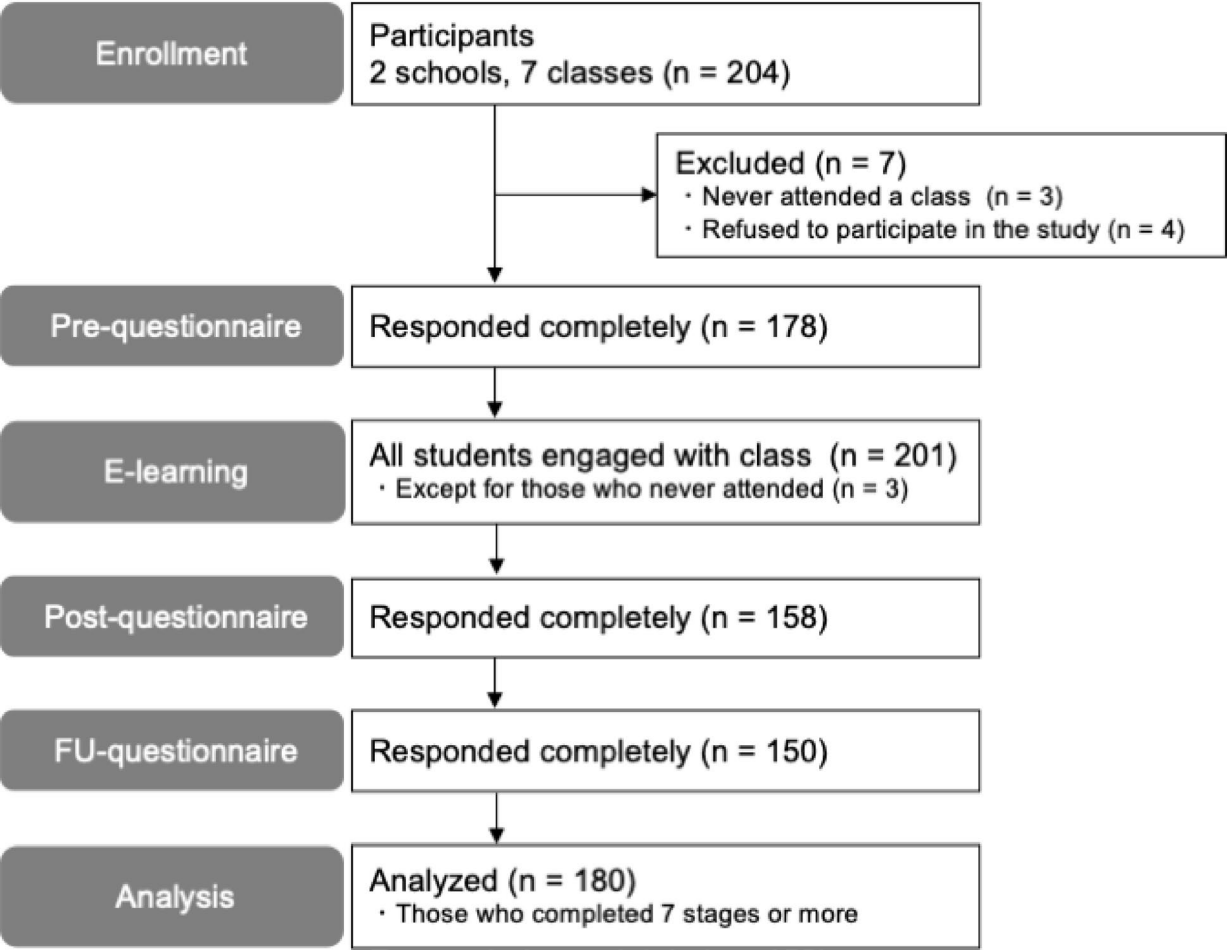
Flowchart displaying the number of participants at each time point

Of the two classes at School A (30 students) and five classes at School B (174 students), three students at School B never attended a class and four students refused to participate in this study; thus, the final number of participants was 197. The gender distribution was as follows: 83 boys, 104 girls, 10 students with inconsistent gender responses, and no students who consistently identified as “other” across all time points. Of these, 76 boys, 95 girls, and 9 students with unknown gender (180 students in total, representing 91.4% of the sample) completed at least seven stages of the program, covering 80% of the total content, and were included in the analysis (Fig. 1). The mean baseline (pre-intervention) anxiety scores were 21.9 for boys, 32.3 for girls, and 31.2 for students with unknown gender. The baseline scores for School A and School B were 28.6 and 28.0, respectively (Table 1). A Kruskal–Wallis test comparing baseline scores revealed a significant difference across gender groups (*r* =.35, *p* <.01), whereas a Mann–Whitney U test comparing the two schools showed no significant difference in mean scores.

**Table 1:** Mean SCAS baseline scores for those who responded to the pre-questionnaire out of the 180 analyzed

	Number	Mean	Standard deviation
*All respondents*	164	28.06	19.15
*Gender*			
Boys	66	21.88	18.08
Girls	89	32.33	16.09
Unknown gender	9	31.22	37.92
*School*			
A	28	28.57	18.11
B	136	27.96	19.42

**Fig. 2:**
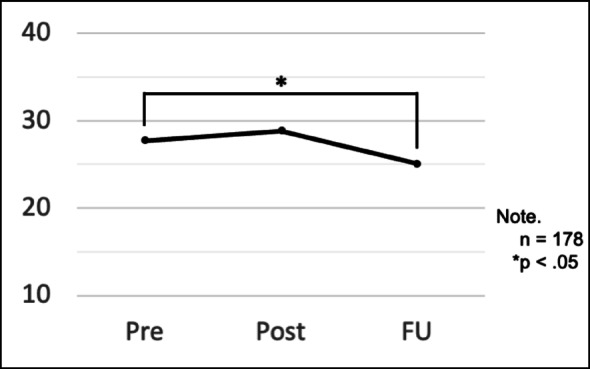
Changes in the estimated mean SCAS scores

The estimated mean scores for the entire SCAS at the three time points based on the GLMM are shown in Table 1. Following Bonferroni’s multiple comparison test, the estimated mean score showed a significant decrease of 2.6 points from pre-follow-up (*F* = 5.56, *p* =.04, 95% CI = (−5.08, −0.09)) (Fig. 2). However, it temporarily increased by 1.14 points between pre-post, with no significant difference (*p =.*59, 95% CI = (−1.31, 3.60)).

**Table:** The estimated mean scores for the entire SCAS at the three time points based on the GLMM

	Time point	Estimated mean	Standard error	95% Confidence interval	
				Lower limit	Upper limit
All respondents (*N* = 178)*	Pre	27.70	2.26	17.78	26.69
	Post	28.84	2.33	15.94	25.13
	Follow-up	25.11	2.35	11.22	20.48

*The data of two students who did not respond to the SCAS once were not included in the estimations

**Fig. 3:**
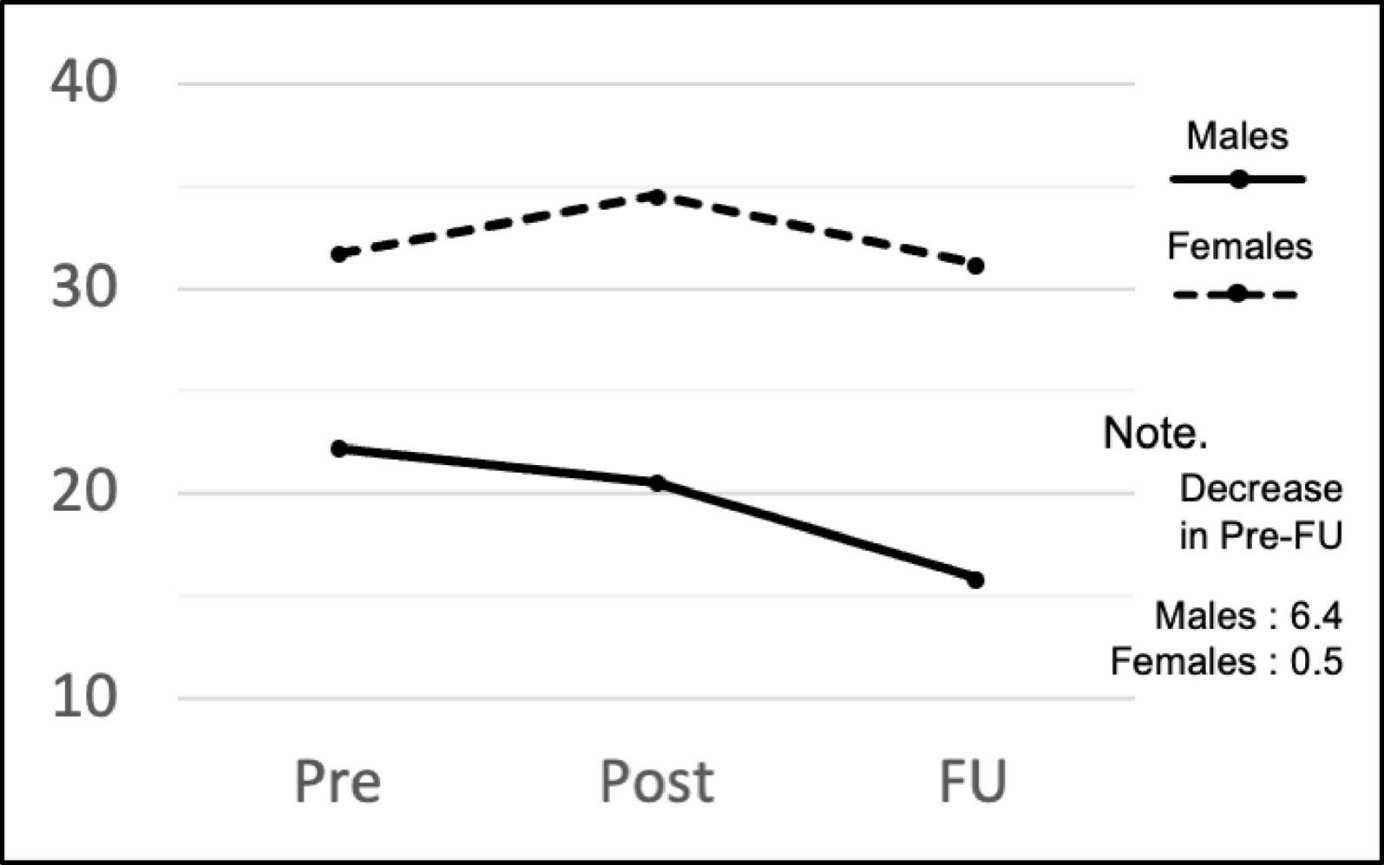
Changes in the estimated mean SCAS scores by gender

**Fig. 4:**
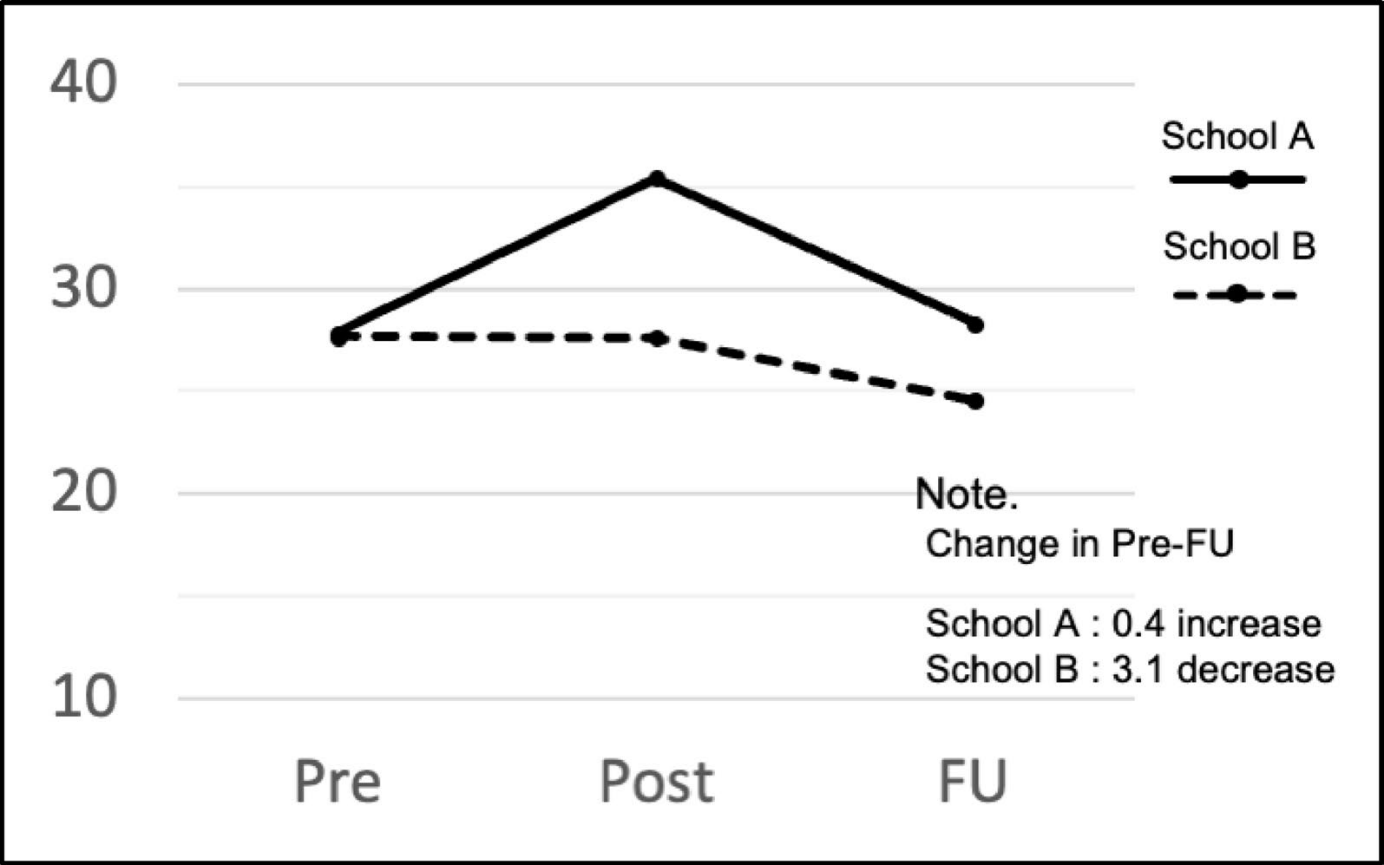
Changes in the estimated mean SCAS scores by school

The estimated mean SCAS scores for boys and girls, as well as for each school at the three time points, are shown in Table 2. For boys, the score decreased by 6.4 points between pre-follow-up, whereas for girls, the score decreased by only 0.5 points (Fig. 3). The score increased by 0.4 points pre-follow-up for School A, whereas the score decreased by 3.1 points for School B (Fig. 4). In the subgroup analysis, the interactions between time and gender (*p =.*03) and between time and school (*p =.*04) were both significant. In this sample, both gender and school differences affected changes in scores over time.

**Table 2:** The estimated mean SCAS scores for boys and girls, and for each school, at the three time points

	Time point	Estimated mean	Standard error	95% Confidence interval	
				Lower limit	Upper limit
*Gender*					
Boys (*n* = 66)	Pre	22.24	2.26	17.78	26.69
	Post	20.54	2.33	15.94	25.13
	Follow-up	15.85	2.35	11.22	20.48
Girls (*n* = 89)	Pre	31.71	1.97	27.83	35.60
	Post	34.55	2.01	30.59	38.50
	Follow-up	31.19	2.03	27.20	35.19
*School*					
A (*n* = 28)	Pre	27.86	3.94	20.09	35.62
	Post	35.41	4.08	27.38	43.44
	Follow-up	28.30	4.08	20.27	36.33
B (*n* = 136)	Pre	27.67	1.78	24.16	31.17
	Post	27.61	1.81	24.04	31.17
	Follow-up	24.53	1.83	20.93	28.13

**Fig. 5:**
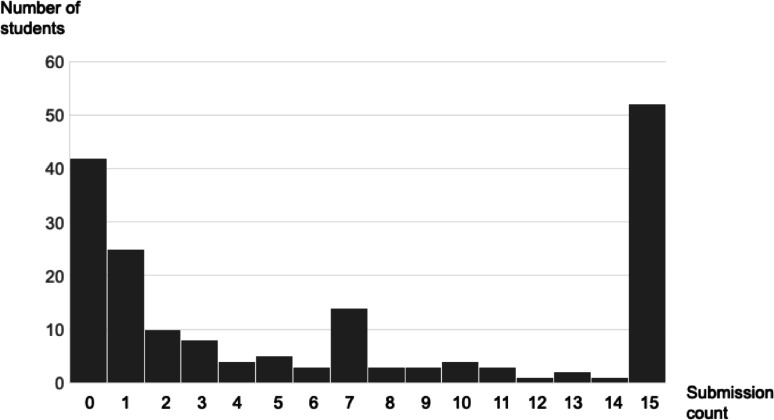
Number of students by homework submission count

The average score for the comprehension test (true/false questions) in the final stage was 13.2 out of 16 (SD = 2.2). The average number of homework submissions was 6.6 out of a maximum of 15 (SD = 6.2), with the most frequent number of submissions being 15 (52 students, 28.9%), and the next highest being 0 (42 students, 23.3%) (Fig. 5).

**Fig. 6:**
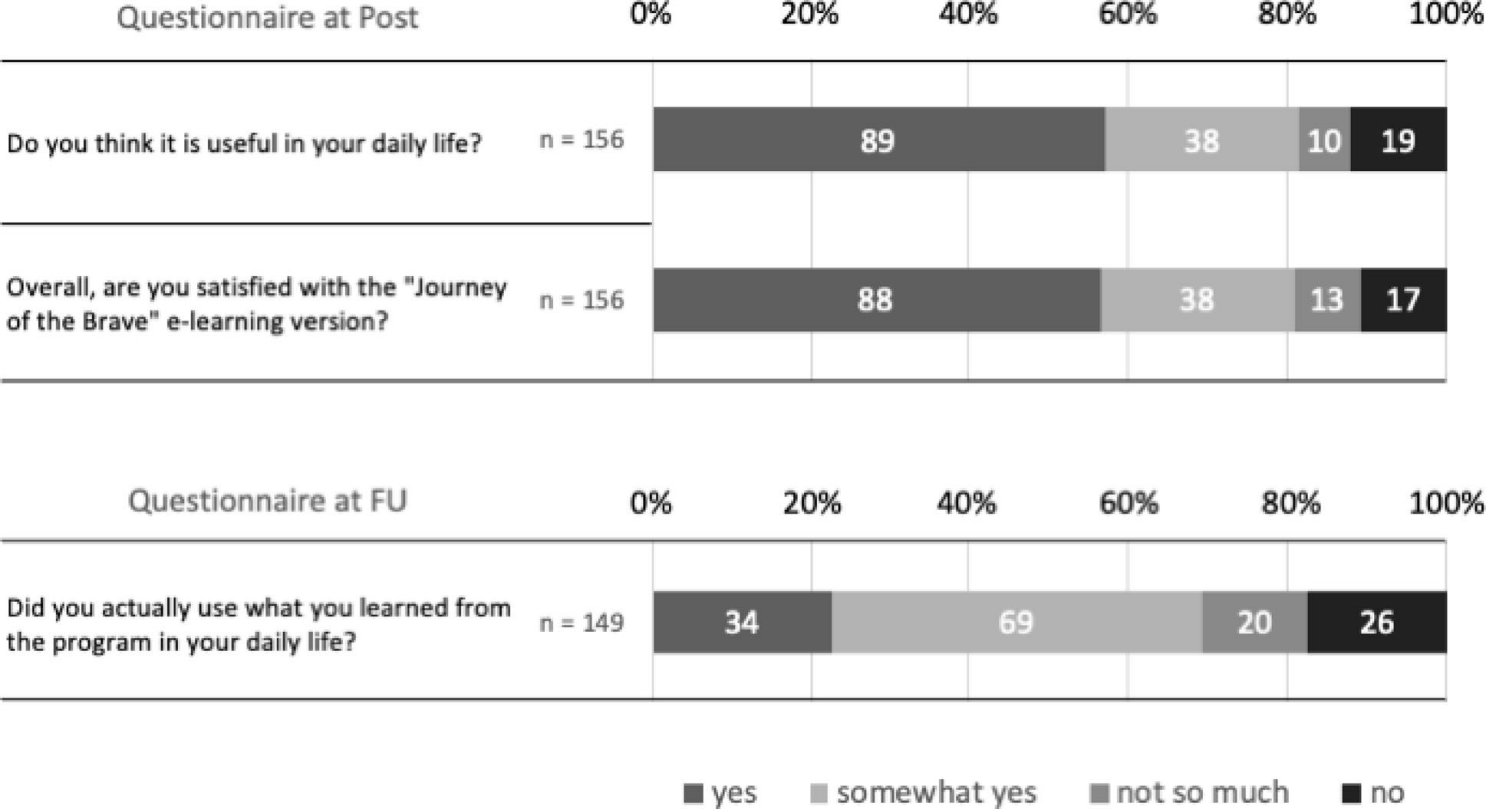
Results of the satisfaction questionnaire at post and follow-up

In the satisfaction questionnaire at the post-follow-up measurement, 81% of the students responded “yes” or “somewhat yes” to the questions “Do you think it is useful in your daily life?” and “Overall, are you satisfied with the ‘Journey of the Brave’ e-learning version?” Additionally, 69% of the students responded “yes” or “somewhat yes” to the question “Did you actually use what you learned from the program in your daily life?” at the follow-up (Fig. 6).

## Discussion

### Changes in anxiety scores

In this study, the estimated mean scores for the entire SCAS decreased significantly from pre-intervention to follow-up. This is similar to the results of our previous study, Ohashi et al. [20], where the scores also decreased from pre-follow-up, although there was no significant difference. According to subgroup analysis, this trend was inconsistent across gender and schools. Since this study was a single-arm intervention with no control group, the aim was not to evaluate the anxiety-reducing effects. However, the overall downward trend in anxiety scores supports the feasibility of implementing anxiety-prevention programs through e-learning in school settings to some extent. Following the findings of our previous study [20], education on understanding emotions may have improved emotion regulation skills and, subsequently, reduced anxiety. We also believe that exposure to anxiety, an effective element of CBT [6], may have been enhanced because e-learning facilitated the practice of certain skills that we describe below.

In contrast, the finding that gender and school affected score changes has not been mentioned in previous studies [11–13] exploring the “Journey of the Brave” as a face-to-face program. It is possible that the boys preferred the game-like system design of the e-learning version, such as collecting points, which increased the effectiveness of the e-learning method compared to face-to-face learning. However, since these data are from treatment intervention studies, it will be necessary to examine whether e-learning is more effective in reducing anxiety among boys in the context of prevention; there is no evidence to suggest that gender predicts the effectiveness of cognitive behavioral therapy interventions [24, 25]. It is more likely that the differences in gender and school were due to the varying groups of students with whom participants spent time. This is because the relationships among students in each school, and even in each class, differ. In Japanese elementary schools, homeroom teachers are typically responsible for teaching almost all subjects except for specialized areas such as music and art. Thus, the e-learning version “Journey of the Brave” was also taught by these same teachers in each class. Consequently, the relationship between teachers and children may have differed from class to class. Additionally, some classes at School B were suspended due to an influenza epidemic during the study period, resulting in significant schedule changes and delays of up to two months in the completion of all nine classes. The epidemic itself, the period during which students could not attend class, and the anxiety felt by teachers and students regarding the delayed classes may have contributed to the anxiety scores. Additionally, the timing of the questionnaires, which were administered during winter and spring breaks or just before graduation, as well as school events and the environment at that time (for example, year-end report cards, graduation ceremonies, or changes in next year’s classes), may have affected the changes in anxiety scores. It will be important to consider the effect of differences in the learning environment when evaluating the effectiveness of the program in the future.

### Retention of learning and satisfaction

In this study, the students’ average score on the comprehension test was 13.2 points out of 16 points (*SD* = 2.2), lower than the average score of 14.6 points in Ohashi et al. [20]. However, unlike in Ohashi et al. [20], where only those who wished to participate were included, the average score of over 80% indicates that the “Journey of the Brave” e-learning version contributed to the acquisition of knowledge that helps prevent anxiety even when implemented in a school setting. Regarding homework, the average number of submissions was 6.6 out of a maximum of 15, and the results were split into two extremes: students who completed all the homework and those who did not complete any, similar to Ohashi et al. [20]. For a universal prevention program that does not always assume participants have anxiety problems, it may be expected for the results to be divided between these two extremes. Regarding prevention, it may be more important to focus on whether the cognitive behavioral therapy-based know-how learned through the program has been incorporated into the students’ lives rather than on whether students continue to do their homework during the study period. From this perspective, the fact that around 70% of the students responded that they “actually used what they learned from the program in their daily lives” during the follow-up (conducted some time after the last e-learning session) also indicates that the e-learning contributed to retention.

Furthermore, although the level of satisfaction was slightly lower than that in Ohashi et al. [20], more than 80% of the students still indicated that the program was useful and positively evaluated their participation. These results show that the “Journey of the Brave” e-learning version is acceptable and feasible for children, even when implemented in schools. In Japanese elementary schools, homeroom teachers are typically responsible for teaching most subjects. Consequently, it can be a considerable burden for them to relearn specialized topics like mental health and provide nine class hours on such themes. Therefore, it is practically important to offer children access to anxiety-prevention knowledge and skills in a way that minimizes teacher workload, including preparation and assessment tasks. Since the students who participated in the e-learning program had not previously experienced the face-to-face “Journey of the Brave” sessions, it remains unclear whether they preferred the e-learning format over the traditional method. Future studies should explore this aspect to better understand the advantages of implementing the e-learning version.

### Limitations and future issues

While this study makes several valuable contributions, some limitations should be considered. First, this was a single-arm intervention study; thus, we cannot assert that the reduction in the estimated mean SCAS score from pre-intervention to follow-up was due solely to e-learning. Additionally, the information we received was limited to grade, gender, e-learning completion history, and questionnaire responses regarding SCAS and satisfaction. All other information was anonymized. This meant that we were unable to consider various factors that could affect anxiety scores, such as regular school attendance outside of e-learning, visits to the infirmary, a history of counseling with school counselors, and problem behaviors. Based on the results of this study, which was the first to verify the feasibility of implementing the “Journey of the Brave” e-learning program in schools, a comparative study using more detailed information on the participants and including a control group should be conducted in the future to clarify the effect of the intervention. This study also showed that gender and school can impact the results. When evaluating the effectiveness of a program through a comparative study between groups, it is necessary to analyze not only whether the overall average score improves but also which factors, such as the demographics of the students and the learning environment, influence effectiveness. This will enable the consideration of better methods to promote anxiety-prevention education for children.

There were limitations not only in the research design but also in the evaluation scales used. We were only able to use the SCAS, a self-evaluation scale for children, as a psychological tool because we had to conduct the questionnaire within a limited class time. Thus, we were unable to conduct a multidimensional evaluation that included assessments from others, as in Ohashi et al. [20]. In an interview with the teachers after the follow-up, we received feedback from School A, where the SCAS score increased, that “the students’ behavior has changed” and “we feel that it was very effective.” To assess changes in children’s anxiety more accurately, it is necessary to use objective measures. It may also be possible to examine other aspects thought to contribute to future mental health, such as emotion regulation skills—which were the focus of a previous study—while considering interventions as preventive measures for groups that include children who do not necessarily have anxiety problems. In the next phase of research, which should evaluate the effectiveness of the intervention in the “Journey of the Brave” e-learning version, it is necessary to reconsider measures for assessing prevention effects, as well as methods for collecting the necessary data within the limited class time.

## Conclusion

In this study, we evaluated the feasibility, acceptability, and practical issues of the “Journey of the Brave” e-learning program based on cognitive behavioral therapy by measuring changes in anxiety scores, satisfaction, and learning logs among 180 students enrolled in two elementary schools. The students’ anxiety scores significantly decreased from pre-intervention to follow-up. Additionally, students could acquire knowledge and skills related to anxiety prevention through e-learning that they could incorporate into their lives. These results demonstrate that the e-learning version of the “Journey of the Brave” is feasible and acceptable for implementation in schools. This study also suggests that children’s demographics and learning environment may affect changes in anxiety scores. In the future, it is necessary to evaluate the preventive effect of the program through comparative studies with a control group and to clarify the factors that affect the results.

## Data Availability

No datasets were generated or analysed during the current study.

## Abbreviations

CBT: Cognitive behavioral therapy
I-CBT: Internet-based cognitive behavioral therapy
SCAS: Spence Children’s Anxiety Scale
MEXT: Ministry of Education, Culture, Sports, Science and Technology
GLMM: Generalized linear mixed model
